# Ginsenoside Rb1 Prevents MPP^+^-Induced Apoptosis in PC12 Cells by Stimulating Estrogen Receptors with Consequent Activation of ERK1/2, Akt and Inhibition of SAPK/JNK, p38 MAPK

**DOI:** 10.1155/2012/693717

**Published:** 2012-09-16

**Authors:** Ryo Hashimoto, Jing Yu, Hideki Koizumi, Yasuyoshi Ouchi, Tetsuro Okabe

**Affiliations:** ^1^Department of Integrated Traditional Medicine, Graduate School of Medicine, The University of Tokyo, 7-3-1 Hongo, Bunkyo-ku, Tokyo 113-0033, Japan; ^2^Department of Geriatric Medicine, Graduate School of Medicine, The University of Tokyo, 7-3-1 Hongo, Bunkyo-ku, Tokyo113-0033, Japan

## Abstract

Ginsenoside Rb1 shows neuroprotective effects in various neurons, including dopaminergic cells. However, the precise mechanisms of action are uncertain. In this paper, we examine whether Rb1 has a neuroprotective effect on MPP^+^-induced apoptosis and attempt to clarify the signaling pathway in PC12 cells. Apoptosis of PC12 cells was determined by DNA fragmentation assay, the activation of caspase-3, or by the inactivation of Bcl-xL. Rb1 inhibited MPP^+^-induced caspase-3 activation and DNA fragmentation and activated Bcl-xL in MPP^+^-treated PC12 cells. These antiapoptotic effect was abrogated in PC12 cells transfected with estrogen receptor siRNA. Levels of DNA fragmentation were increased by wortmannin or PD 98059, while they were decreased by SB 203580 or SP 600125 in MPP^+^-treated PC12 cells. Rb1 increased phosphorylation levels of ERK1/2 or Akt in MPP^+^-treated PC12 cells, while it reduced phosphorylated p38 or SAPK/JNK. The increased phosphorylation of ERK/1/2 or Akt by Rb1 was abrogated by estrogen receptor siRNA. Rb1-induced inhibition of SAPK/JNK or p38 phosphorylation was also abolished by estrogen receptor siRNA. These results suggest that ginsenoside Rb1 protects PC12 cells from caspase-3-dependent apoptosis through stimulation of estrogen receptor with consequent activation of ERK1/2 and Akt and inhibition of SAPK/JNK and p38.

## 1. Introduction 

**Figure 1:**
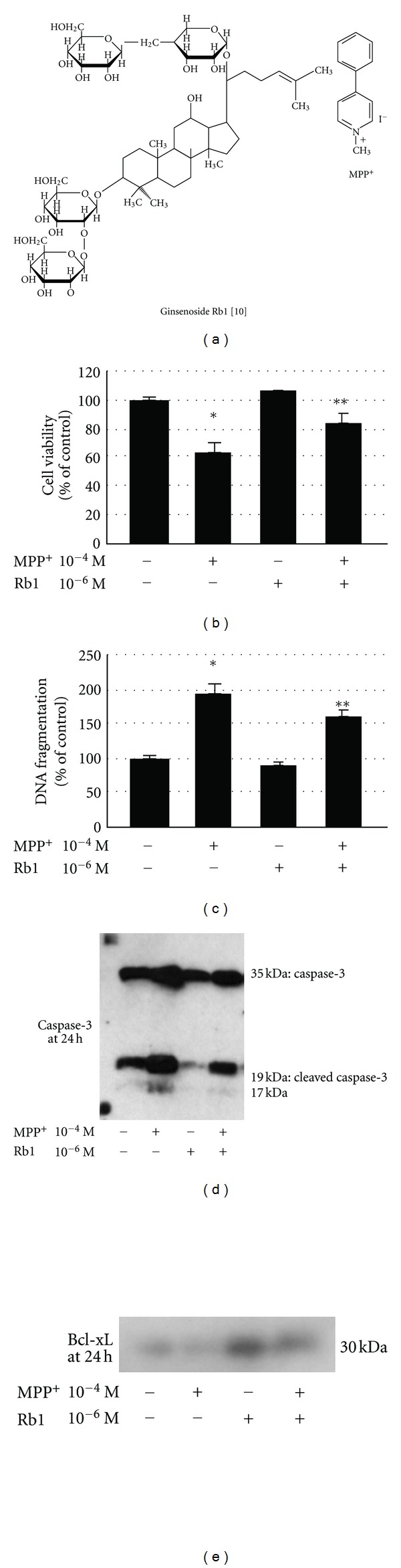
(a) Structure of ginsenoside Rb1 and MPP^+^. (b) Protective effects of ginsenoside Rb1 on MPP^+^-induced cytotoxicity. PC12 cells were pretreated with 10^−6^ M ginsenoside Rb1 for 4 hours and then exposed to 10^−4^ M MPP^+^ for 24 hours. The cell viability was expressed as the percent (%) of vehicle value by resazurin reduction (CellTiter-Blue) assay. *P < 0.01 versus vehicle, **P < 0.05 versus MPP^+^ treated group. (c) DNA fragmentation of PC12 cells induced by MPP^+^. PC12 cells were pretreated with 10^−6^ M ginsenoside Rb1 for 4 hours and then exposed to 10^−4^ M MPP^+^ for 24 hours. The experimental values were normalized and presented as a percentage of the vehicle section. The data represent means ± SD of three independent experiments. *P < 0.01 versus vehicle, **P < 0.05 versus MPP^+^ treated group. (d, e) Representative blots illustrating the effects of ginsenoside Rb1 during MPP^+^ exposure on activation of caspase-3 and Bcl-xL in PC12 cells. Cells were pretreated with 10^−6 ^M ginsenoside Rb1 for 4 hours and then exposed to 10^−4 ^M MPP^+^ for 24 hours.

Ginseng (botanical name: *Panax ginseng C.A.Meyer*) is one of the most popular herbal remedies and ginseng root extracts have long been used in traditional Chinese medicine to restore and enhance well-being [1]. Recent studies have suggested that administration of *Panax ginseng* powder improved cognitive performance in patients with Alzheimer disease [2], and in healthy young volunteers [3]. Ginseng also ameliorated psychomotor performance during exercise without affecting exercise capacity [4] and improved certain psychomotor functions in healthy subjects [5]. Ginsenosides, saponins of *Panax ginseng*, are structurally similar to steroids (Figure 1(a)) [6].*Panax ginseng* and ginsenosides are reported to attenuate degeneration of dopaminergic neurons and symptoms in vitro and in vivo conditions [7]. Recent studies have demonstrated the neuroprotective effect by ginsenoside Rb1, a major active component of the traditional herb ginseng, on some experimental models [8–10]. 

Defects in complexes I, II, and IV of the mitochondrial respiratory chain have been detected in Alzheimer, Parkinson, and Huntington diseases [11]. 1-Methyl-4 phenylpyridinium (MPP^+^), an active toxic metaboliteof1-methyl-4-phenyl-1,2,3,6-tetrahydropyridine (MPTP), is selectively accumulated in dopaminergic neurons and is concentrated within mitochondria where it acts to inhibit electron transport chain, decreases mitochondrial membrane potential, and induces disturbances in Ca^2+^ homeostasis, which could eventually lead to neuronal loss [12]. 

PC12 cells, a clonal rat pheochromocytoma cell line, possess dopamine synthesis, metabolism, and transporting systems and therefore have been used extensively as a model for studies of MPP^+^ neurotoxicity and Parkinson disease [13].

 Aim of the present work was to clarify intracellular signaling pathways involved in ginsenoside-Rb1-induced cytoprotective effects on MPP^+^ toxicity in PC 12 cells.

## 2. Materials and Methods

### 2.1. Materials

Ginsenoside-Rb1 standard was obtained from Wako Pure Chemical Industries, Ltd. (Osaka, Japan). 1-Methyl-4 phenylpyridinium (MPP^+^) iodine was purchased from Sigma-Aldrich, Inc. (St Louis, MO, USA). The structures of ginsenoside Rb1(2-*O*-**β**-Glucopyranosyl-(3**β**,12**β**)-20-[(6-*O*-**β**-D-glucopyranosyl-**β**-D-glucopyranosyl)oxy]-12-hydroxydammar-24-en-3-yl *β*-D-glucopyranoside) and MPP^+^ are illustrated in Figure 1(a). Cell Death Detection ELISA Plus kit was from Roche Diagnostics GmbH (Penzberg, Germany). Celltiter-blue Cell Viability Assay was purchased from Promega (Madison, WI, USA). Wortmannin, SP 600125, and SB 203580 were from Calbiochem (Merck KGaA, Darmstadt, Germany). PD 98059 was obtained from Santa Cruz biotechnology (Santa Cruz, CA, USA). Estrogen receptor (ER) alpha and beta small interfering RNA (siRNA) were from Santa Cruz biotechnology. Androgen receptor (AR) siRNA was from Sigma-Aldrich. Phospho-Akt, Akt, phospho-p44/42 MAPK (ERK1/2), ERK1/2, phospho-SAPK/JNK, SAPK/JNK, caspase-3, and Bcl-xL antibodies were purchased from Cell Signal Technology, Inc. (Beverly, MA, USA). Phospho-p38 MAPK and p38 MAPK antibodies were obtained from Santa Cruz biotechnology. Dulbecco's modified Eagle's medium (DMEM) was purchased from Sigma-Aldrich. Fetal bovine serum (FBS) was obtained from Sanko Junyaku Co., Ltd. (Tokyo, Japan). Horse serum (HS) was from LifeTechnologies (Carlsbad, CA, USA).

### 2.2. Cells Culture and Treatments

 Undifferentiated rat pheochromocytoma PC12 cells were cultured in 75 cm^2^ tissue culture flasks (BD Falcon, CA, USA). Cells were maintained in Dulbecco's modified Eagle's medium (DMEM) containing 5% heat-inactivated FBS and 10% heat-inactivated HS supplemented with 100 U/mL penicillin and 100 *μ*g/mL streptomycin in a water-saturated atmosphere of 5% CO_2_ in air at 37 degrees centigrade. Cells were plated in 60 mm dishes (for Western blot analysis) or 24-well culture plate (for cell viability assay and apoptotic DNA fragmentation assay) at a density of 0.5×10^6^ cells/cm^2^ and grown to 90% confluency. Cells were stabilized for 24 hours in serum-free medium. Ginsenoside Rb1 Standard, MPP^+^, ER alpha, beta, AR siRNA were added to PC12 cells for indicated time described elsewhere. 

### 2.3. Cell Viability Assay

 Cell viability was assayed with CellTiter-Blue Cell Viability Assay (Promega, Madison, WI, USA). This assay evaluates metabolic capacity of viable cells to reduce resazurin into resorufin, which is highly fluorescent. Nonviable cells do not generate fluorescent signal to lose ability to reduce resazurin. Assays were performed according to the manufacturer's instructions. Briefly, PC12 cells were seeded at a density of 0.5×10^6^ cells/cm^2^ in 24-well plates and grown to 90% confluency. PC12 cells were pretreated with or without 10^−6^ M ginsenoside Rb1 for 4 hours and then exposed to 10^−4 ^M MPP for 24 hours. After the incubation period, 20 *μ*L CellTiter-Blue reagent (highly purified resazurin) was added to each well. The plates were incubated 37 degrees centigrade for 4–6 hours. The amount of fluorescence was quantitated on Bio-Rad model 550 Microplate Reader (Bio-Rad Laboratories, Inc., Hercules, CA, USA) at 590 nm. 

### 2.4. Measurement of Apoptotic Cell Death (DNA Fragmentation Assay)

Apoptotic cell death was assayed with Cell Death Detection ELISA plus (Roche Diagnostics GmbH, Penzberg, Germany). This assay is a quantitative sandwich enzyme immunoassay using mouse monoclonal antibodies directed against DNA and histones, respectively, and evaluates cytoplasmic histone-associated DNA fragments after induced cell death. Assays were performed according to the manufacturer's instructions. PC12 cells were seeded at a density of 0.5×10^6^ cells/cm^2^ in 24-well plates and grown to 90% confluency. PC12 cells were pretreated with or without 10^−6^ M ginsenoside Rb1 for 4 hours and then exposed to 10^−4^ M MPP^+^ for 24 hours. The fluorescence was recorded on Bio-Rad model 550 microplate reader (Bio-Rad Laboratories, Inc., Hercules, CA, USA) at 405 nm. The final absorbance was calculated by subtracting the absorbance of the untreated cells and background value. Each sample was analyzed in triplicate.

### 2.5. Western Blot Analysis

 Cultured confluent monolayers of cells were lysed in a buffer containing 20 mmol/L Tris-HCl (pH 7.5), 150 mmol/L NaCl, 1 mmol/L EDTA, 1 mmol/L EGTA, 1% Triton-X, 2.5 mmol/L sodium pyrophosphate, 1 mmol/L *β*-glycerophosphate, 1 mmol/L Na3VO4, 1 *μ*g/mL leupeptin, and 1 mmol/L PMSF. Total cell lysates containing indicated amounts of protein were electrophoresed in 12.5% SDS-PAGE and then transferred to polyvinylidene difluoride (PVDF) membrane. The membranes were blocked with 5% defatted milk and were washed in phosphate-buffered saline containing 0.1% Tween20 (TBST). The membranes were incubated with anti-caspase-3 (1 : 1000 dilution), anti-Bcl-xL (1 : 1000 dilution), anti-phosphospecific p38 MAPK (1 : 400 dilution), anti-p38 MAPK (1 : 400 dilution), anti-phosphospecific SAPK/JNK (1 : 2000 dilution), anti-SAPK/JNK (1 : 2000 dilution), anti-phosphospecific ERK1/2 (1 : 10000 dilution), anti- ERK1/2 (1 : 10000 dilution), anti-phosphospecific Akt (1 : 1000 dilution), anti-Akt antibodies (1 : 1000 dilution) overnight at 4 degrees centigrade. The membranes were washed three times for 10 minutes each in TBST, after which they were incubated for 1 hour with anti-rabbit or anti-mouse IgG horseradish peroxidase conjugated secondary antibodies (1 : 1000 dilution). The membranes were washed three times for 20 minutes each in TBST. Blots were developed using luminol chemiluminescent substrate (LumiGLO Kirkegaard & Perry Laboratories, Inc., Gaithersburg, MD, USA) and detected using photographic film (KODAK Biomax Film).

### 2.6. Small Interfering RNA Transfection

siRNA-inhibited ER alpha or beta were used separately. PC12 cells were seeded at a density of 0.5×10^6^ cells/cm^2^ in 60 mm dishes and grown to 60% confluency in nonantibacterial medium. Then cells were transfected with 10 nM ER alpha or beta siRNA using HiperFect transfection reagent (Qiagen) in 1 mL transfection medium (Santa Cruz). Two hours later, 3 mL of culture medium was added to each dish for another 22 hours. Knockdown effect was assessed by Western blot using ER alpha or beta antibodies. 

### 2.7. Statistical Analysis

 Data are expressed as mean ± S.D. values. Statistical significance was assessed by the one-way analysis of variance (ANOVA). Probability values less than 0.05 were considered statistically significant.

## 3. Results

### 3.1. Ginsenoside Rb1 Affords Neuroprotective Effect against MPP^+^-Induced Cytotoxicity in PC12 Cells

Cell viability decreased to 65% of vehicle in cells exposed to 10^−4^ M MPP^+^ for 24 hours. Ginsenoside Rb1 significantly improved cell viability in MPP^+^-treated PC12 cells (Figure 1(b)). We also assayed DNA fragmentation. PC12 cell apoptosis induced by MPP^+^ was significantly attenuated by treatment with ginsenoside Rb1 (Figure 1(c)). 

### 3.2. Ginsenoside Rb1 Induces Downregulation of Caspase-3 and Upregulation of Bcl-xL

The activities of caspase-3 and Bcl-xL were determined by Western blot analysis. The Bcl-2 family member Bcl-xL is related to apoptosis resistance by heterodimerization with an apoptotic protein to inhibit its apoptotic effect and by its direct pore-forming effect on the outer membrane of mitochondria to help maintain a normal membrane state under stress conditions [14–17]. When PC12 cells were treated with ginsenoside Rb1 alone, the activity of caspase-3 was less than that seen in vehicle. Exposure to MPP^+^ increased the activity of caspase-3 compared to vehicle. Treatment with ginsenoside Rb1 reduced the activity of caspase-3 in MPP^+^-treated PC12 cells (Figure 1(d)). When treated with ginsenoside Rb1 alone, PC12 cells showed greater activity of Bcl-xL than that seen in vehicle. MPP^+^ reduced the activity of Bcl-xL compared to vehicle and addition of both MPP^+^ and ginsenoside Rb1 increased the activity of Bcl-xL compared to MPP^+^ alone group (Figure 1(e)).

### 3.3. Antiapoptotic Effect by Ginsenoside Rb1 Was Abolished with Estrogen Receptor Alpha or Beta siRNA, but Not with Androgen Receptor siRNA

**Figure 2:**
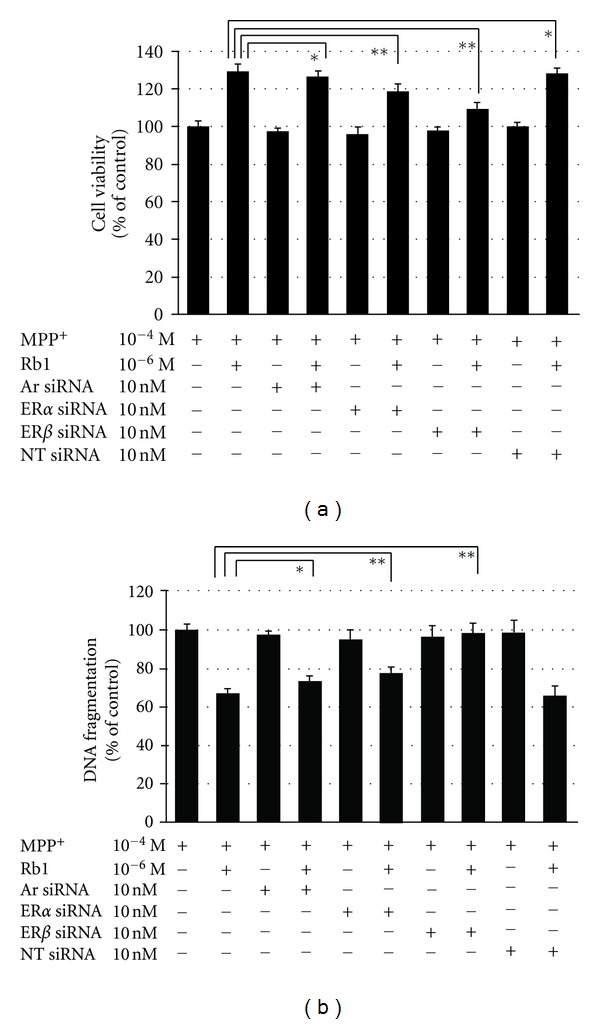
Antiapoptotic effect by ginsenoside Rb1 was abolished by ER alpha or beta siRNA but not by AR siRNA. Cells were transfected with 10 nM ER-alpha or -beta siRNA using HiperFect transfection reagent in 1 mL transfection medium for two hours. Then 3 mL of culture medium was added to each dish for another 22 hours. (a) The improvement of cell survival by ginsenoside Rb1 was abolished by ER alpha or beta siRNA, but not by AR siRNA; *not significant, **P < 0.05. (b) The restraint of DNA fragmentation by ginsenoside Rb1 was abolished by ER alpha or beta siRNA, but not by AR siRNA; *not significant, **P < 0.05.

In order to investigate which steroid receptors would be involved in the Rb1-mediated antiapoptotic action, PC12 cells were transfected with 10 nM ER alpha or beta siRNA using HiperFect transfection reagent in 1 mL transfection medium for two hours. Then 3 mL of culture medium was added to each dish for another 22 hours. PC12 cells were treated with 10^−6^ M ginsenoside Rb1 for 4 hours prior to exposing PC12 cells to 10^−4^ M MPP^+^ for 24 hours. The results showed that the improvement of cell survival or the restraint of DNA fragmentation induced by ginsenoside Rb1 was abrogated by ER alpha or beta siRNA but not by AR siRNA (Figures 2(a) and 2(b)). 

### 3.4. Ginsenoside Rb1 Activates the Phosphorylation of Akt, ERK1/2 and Inactivates the Phosphorylation of SAPK/JNK, P38 MAPK

**Figure 3:**
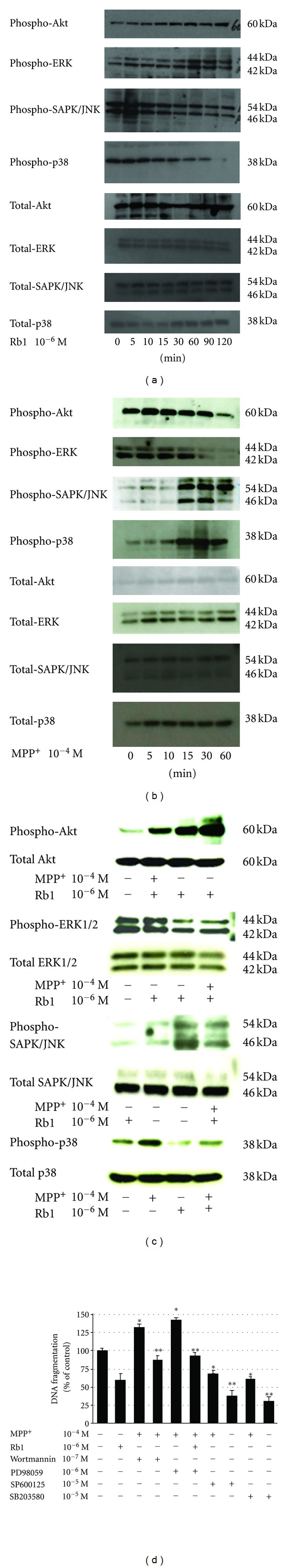
(a) Time course during ginsenoside Rb1 exposure in PC12 cells to phosphorylation of Akt, ERK1/2, SAPK/JNK, or p38 MAPK by Western blot. (b) Time course of phospho-p38 MAPK, phospho-SAPK/JNK, phospho- ERK1/2, or phospho-Akt protein expression during MPP^+^ exposure in PC12 cells by Western blot. Total protein expression of p38 MAPK, SAPK/JNK, ERK1/2, or Akt was also shown. (c) Effects of ginsenoside Rb1 in MPP^+^-mediated apoptotic signaling. PC12 cells were pretreated with 10^−6^ M ginsenoside Rb1 for 1hour and then exposed to 10^−4^ M MPP^+^ for 30 minutes. Total protein expression of p38 MAPK, SAPK/JNK, ERK1/2, Akt are also shown. (d) Effects of wortmannin, PD 98059, SB 203580, or SP 600125 on DNA fragmentation assay. PC12 cells were pretreated with 10^−7^ M wortmannin, 10^−5^ M PD 98059, 10^−5^ M SB 203580, or 10^−5^ M SP 600125 for 1 hour before the addition of 10^−6^ M ginsenoside Rb1 for 4 hours and then exposed to 10^−4^ M MPP^+^ for 24 hours. The experimental values were normalized and presented as a percentage of the vehicle section. The data represent means ± SD of three independent experiments. *P < 0.05 versus MPP^+^ treated group, **P < 0.05 versus MPP^+^ and Rb1 treated group.

 Phosphorylation levels of Akt at serine 473 and ERK1/2 at Thr 202/Tyr 204 were increased and those of SAPK/JNK at Thr 183/Tyr 185 and p38 MAPK at Tyr 182 were reduced within 2 hours after exposure to 10^-6 ^M ginsenoside Rb1 in PC12 cells (Figure 3(a)). Levels of the phosphorylated form of SAPK/JNK or p38 MAPK were increased and those of the phosphorylated forms of Akt or ERK1/2 were reduced within 1hour by MPP^+^ treatment (Figure 3(b)). The results in Figure 3(c) showed the effect of ginsenoside Rb1 on MPP^+^-mediated apoptotic signaling. Ginsenoside Rb1 reduced phosphorylated protein expression of SAPK/JNK or p38 MAPK and increased phosphorylated protein expression of Akt or ERK1/2. 

Levels of DNA fragmentation were increased by wortmannin (a specific and direct inhibitor of PI3 kinase) or PD 98059 (a highly selective inhibitor of MEK1) while it were decreased by SB 203580 (a highly specific p38 MAPK inhibitor) or SP 600125 (an inhibitor of JNK) in MPP^+^ treated PC12 cells (Figure 3(d)).

### 3.5. Estrogen Receptor Is Involved in Ginsenoside Rb1-Induced Activation of Akt, ERK1/2 and Inactivation of SAPK/JNK, p38 MAPK

**Figure 4:**
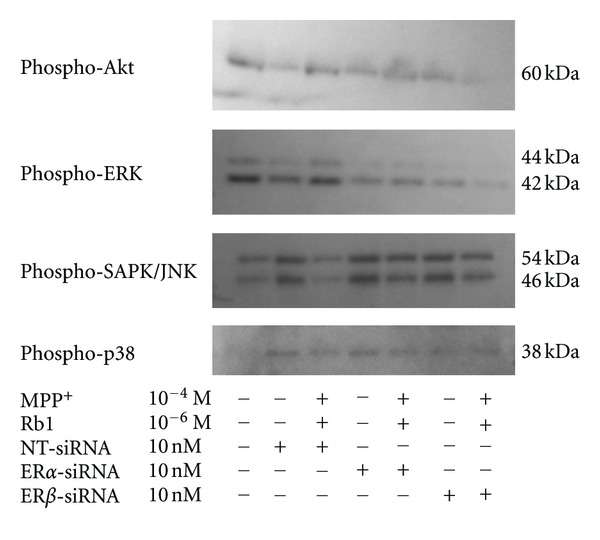
Estrogen receptor involvement in p38 MAPK, SAPK/JNK, ERK1/2, or Akt phosphorylation induced by ginsenoside Rb1 in MPP^+^-treated PC12 cells. Cells were transfected with 10 nM ER-alpha or -beta siRNA using HiperFect transfection reagent in 1 mL transfection medium for two hours. Then 3 mL of culture medium was added to each dish for another 22 hours.

In order to investigate which receptors could be involved in the ginsenoside Rb1-mediated changes in the apoptotic signaling, we used siRNA to knock down gene expression of ER alpha or beta. The increase of phosphorylated forms of Akt, ERK1/2 and the decrease of phosphorylated SAPK/JNK, p38 MAPK induced by ginsenoside Rb1 were significantly abrogated by suppressing expression of ER alpha or beta (Figure 4).

## 4. Discussion

 Ginsenosides with a few exceptions share a similar basic structure, consisting of a saturated 1, 2-cyclopentanoperhydrophenanthrene (sterane or gonane) steroid nucleus [18]. The potential health effects of ginsenosides include anticarcinogenic, immunomodulatory, anti-inflammatory, antiallergic, antiatherosclerotic, antihypertensive, and antidiabetic effects, as well as antistress activity and effects on the central nervous system [18]. Although the extracts from fresh or processed *P. ginseng* have been extensively tested for their pharmacological effects, the precise biological functions and underlying action mechanisms of pure molecules from *ginseng* extract are still largely unknown [19]. 

 In this paper we have demonstrated that purified ginsenoside Rb1 has a neuroprotective effect on MPP^+^-induced apoptosis. Ginsenoside Rb1 inhibited MPP^+^-induced caspase-3 activation and cell DNA fragmentation. It also activated Bcl-xL in MPP^+^-treated PC12 cells. 

Interestingly, the antiapoptotic effect by ginsenoside Rb1 was abolished by ER siRNA but not by AR siRNA. 

Ginsenosides (except Ro) belong to a family of steroids named steroidal saponins [20]. It has been demonstrated that ginsenoside Rb1 acts both as phytoestrogen [21–23] and as phytoandrogen [24]. The brain is a highly estrogen responsive tissue where estrogens induce several beneficial actions [25]. Our data indicates that ginsenoside Rb1 possesses estrogenic properties in PC12 cells, suggesting that ginsenoside Rb1 acts as a phytoestrogen in neural tissue.

 Accumulating reports provide the evidence that phosphorylation of SAPK/JNK, p38 MAPK serves as a proapoptotic factor and phosphorylation of ERK1/2, Akt acts as a cell survival factor [6, 13, 26–29]. In this paper, we have demonstrated that the ginsenoside Rb1-induced neuroprotective effects were mediated through the reduction in phosphorylated SAPK/JNK, p38 MAPK and the increase in phosphorylated protein of Akt, ERK1/2. The increased phosphorylation of ERK/1/2 or Akt was abrogated by ER alpha or beta siRNA but was not affected by AR siRNA. Ginsenoside Rb1-induced inhibition of SAPK/JNK or p38 MAPK phosphorylation was also abolished by ER alpha or beta siRNA but not by AR siRNA. Taken together, our findings suggest that ginsenoside Rb1 protects PC12 cells from caspase-3-dependent apoptosis induced by MPP^+^ through stimulation estrogen receptor alpha and beta with the consequent activation of ERK1/2 or Akt and the inhibition of SAPK/JNK or p38 MAPK.

In this study, we have used both DNA fragmentation and caspase activity for measurements of apoptotic cell death, however other specific assays including Terminal deoxynucleotidyl transferase dUTP nick end labeling assay would be required further to confirm the conclusions.

Liu et al. previously demonstrated the absorption and disposition of ginsenosides after oral administration of *Panax notoginseng* extract to rats [30]. According to their reports, the maximal plasma concentrations of the ginsenoside Rb1 were 1000 to 1200 nM after oral administration of *Panax notoginseng* extract within 6 to 10 hours. In our studies, ginsenoside Rb1 showed significant neuroprotective effects at concentrations of 10^−6^ M. Plasma concentration of 10^−6^ M ginsenoside Rb1 has been retained after usual dose of ginseng in human, suggesting a possible mechanism of action by which ginseng has exerted its pharmacological effects in traditional Chinese medicine [1–5].
